# Toll-Like Receptors, Inflammation, and Calcific Aortic Valve Disease

**DOI:** 10.3389/fphys.2018.00201

**Published:** 2018-03-12

**Authors:** Carmen García-Rodríguez, Iván Parra-Izquierdo, Irene Castaños-Mollor, Javier López, J. Alberto San Román, Mariano Sánchez Crespo

**Affiliations:** ^1^Instituto de Biología y Genética Molecular, CSIC-Universidad de Valladolid, Valladolid, Spain; ^2^Centro de Investigación Biomédica en Red de Enfermedades Cardiovasculares (CIBERCV), Madrid, Spain; ^3^Hospital Clínico Universitario, Valladolid, Spain

**Keywords:** Toll-like receptor (TLR), inflammation, NF-κB, osteogenesis, aortic valve interstitial cell (VIC), calcific aortic valve disease (CAVD)

## Abstract

Inflammation, the primary response of innate immunity, is essential to initiate the calcification process underlying calcific aortic valve disease (CAVD), the most prevalent valvulopathy in Western countries. The pathogenesis of CAVD is multifactorial and includes inflammation, hemodynamic factors, fibrosis, and active calcification. In the development of CAVD, both innate and adaptive immune responses are activated, and accumulating evidences show the central role of inflammation in the initiation and propagation phases of the disease, being the function of Toll-like receptors (TLR) particularly relevant. These receptors act as sentinels of the innate immune system by recognizing pattern molecules from both pathogens and host-derived molecules released after tissue damage. TLR mediate inflammation via NF-κB routes within and beyond the immune system, and play a crucial role in the control of infection and the maintenance of tissue homeostasis. This review outlines the current notions about the association between TLR signaling and the ensuing development of inflammation and fibrocalcific remodeling in the pathogenesis of CAVD. Recent data provide new insights into the inflammatory and osteogenic responses underlying the disease and further support the hypothesis that inflammation plays a mechanistic role in the initiation and progression of CAVD. These findings make TLR signaling a potential target for therapeutic intervention in CAVD.

## Inflammation and calcific aortic valve stenosis

Calcific aortic stenosis is the final step of calcific aortic valve disease (CAVD), a slowly progressive complex process which begins with alterations in the valve leaflets that may lead to the development of left ventricular outflow obstruction (Miller et al., 2011; Rajamannan et al., 2011; Pawade et al., 2015). It has been related to atherosclerosis, inflammation, and hemodynamic factors, but active calcification is a feature characteristic (O'Brien, 2006; Yetkin and Waltenberger, 2009). Its prevalence is high and depends on age, affecting more than 2% of people older than 75 years and 8% of people older than 84 (Roberts and Ko, 2005; Nkomo et al., 2006; Go et al., 2014; Otto and Prendergast, 2014). Once symptoms appear the disease progresses rapidly, and most patients undergo either surgical aortic valve replacement or transcatheter aortic valve implantation. An estimated 42,000 and 65,000 valvular implants are performed per year in Europe and the USA, respectively (Bridgewater et al., 2011; Go et al., 2014; Otto and Prendergast, 2014), thus explaining the social impact and growing healthcare costs associated with CAVD.

CAVD is no longer considered a degenerative process related to aging, but an actively regulated process in which many pathogenetic factors remain unknown (O'Brien, 2006; Rajamannan et al., 2011; Towler, 2013). This change in the paradigm is based on three set of data: (1) epidemiological associations of risk factors and higher prevalence and faster progression of CAVD; (2) histopathologic identification in excised stenotic valves of chronic inflammation features, lipoprotein deposition, renin-angiotensin system components, and molecular mediators of calcification; and 3) elucidation of cell-signaling pathways and genetic factors linked to valve disease pathogenesis (O'Brien, 2006). From a clinical point of view, it would be very important to deepen in the knowledge of the disease and identifying potential novel therapeutic targets to delay its progression. Despite extensive research efforts including several randomized controlled trials (Cowell et al., 2005; Rossebø et al., 2008; Chan et al., 2010), no pharmacotherapy strategies currently exist to prevent or treat CAVD.

During the last decades, many studies have shown the relationship between classical atherogenic risk factors and valve calcification that might explain their frequent coexistence in clinical practice. Clinical factors associated with the development of CAVD found in the Cardiovascular Health Study are similar to the cardiovascular risk factors: older age, male gender, serum lipoprotein and LDL levels, height, hypertension, metabolic syndrome and smoking (Stewart et al., 1997). Even though CAVD and atherosclerosis share some ethiopathological features, recent reports have disclosed significant differences in calcification progression in the late stages and the lack of effect of statins in CAVD progression in the SALTIRE trial (Cowell et al., 2005; O'Brien, 2006; Hjortnaes et al., 2010; Dweck et al., 2013).

The inflammatory response is a hallmark of CAVD, which has been extensively demonstrated (O'Brien, 2006; Coté et al., 2013; Mathieu et al., 2015). In the 90s, some studies identified the presence in aortic valve lesions of macrophages and T lymphocytes and expression of chronic inflammation effector molecules like interleukins (IL)-1 and 2, class II human leukocyte antigen, and HLA-DR. More recently, mast cells, pro-inflammatory cytokines, IL-1β and tumor necrosis factor α have been linked to the disease (O'Brien, 2006; Hjortnaes et al., 2010; Dweck et al., 2013). Other mechanisms include the deposition of the atherogenic lipoproteins LDL and lipoprotein(a) in stenotic aortic valves and the activation of the renin-angiotensin system. Remarkably, the typical calcification during CAVD is considered an inflammation-dependent process (Coté et al., 2013; Dweck et al., 2013). New insights using novel imaging approaches that allow simultaneous visualization of inflammation and early mineralization support the paradigm of inflammation-dependent calcification and reveal that inflammation precedes calcification. This suggests three phases for CAVD progression (Otto, 2008; Aikawa and Otto, 2012; Pawade et al., 2015), inflammation being involved in the initiation and propagation phases, whereas in the last phase, calcification rather than inflammation is predominant.

## TLR, inflammation, and disease

**Figure 1:**
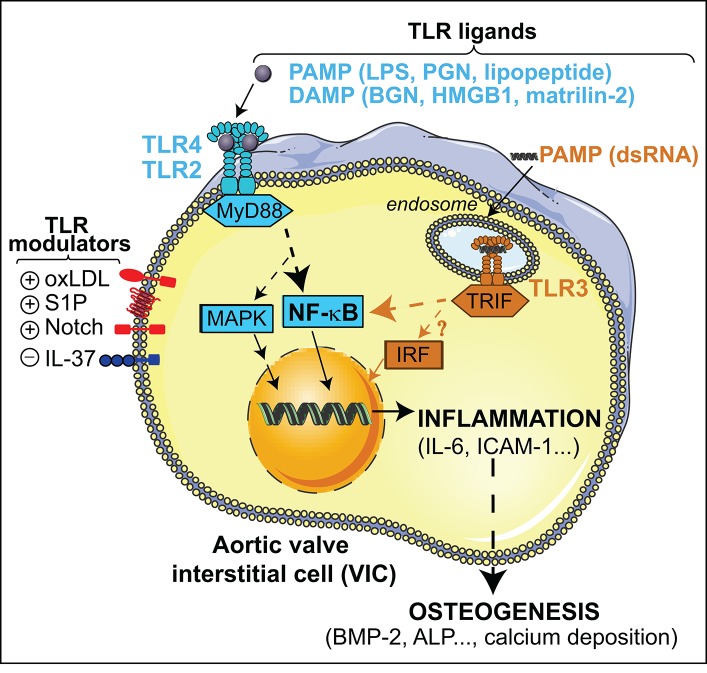
Overview of the TLR signaling pathways triggered by PAMPs and DAMPs in VIC. TLR2/4-MyD88 stimulation leads to NF-κB and MAP kinases activation and the subsequent induction of inflammatory mediators that promotes osteogenic reprogramming of VIC. Endosomal TLR3-TRIF stimulation triggers a non-canonical NF-κB signaling, and most likely IRF activation, thus promoting up-regulation of inflammatory and osteogenic mediators. Putative endosomal TLR4 signaling and TLR4-independent mechanisms are not depicted. ALP, alkaline phosphatase; ICAM-1, Intercellular Adhesion Molecule 1; PGN, peptidoglycan.

The innate immune receptors termed Toll-like receptors (TLR) belong to a phylogenetically ancient system specialized in the recognition of conserved motifs present in pathogens, the so-called pathogen associated molecular patterns (PAMP), and of endogenous molecules released upon tissue injury (DAMP, also known as alarmins) (Kawai and Akira, 2010). The TLR family consists of membrane-spanned receptors recognizing a broad range of ligands, each member sensing a specific set of molecular patterns. So far, 10 TLRs have been identified in humans and 13 in mammalians (Kawai and Akira, 2010). TLR4, the first one described, is the receptor for lipopolysaccharide (LPS), a gram-negative bacterial toxin (Figure 1). TLR2 and its co-receptors TLR1/6 detect lipoproteins and lipopeptides, and TLR5 senses flagellin. TLR2/4 can also sense DAMPs, including heat-shock proteins, high-mobility group box 1 (HMGB1), reactive oxygen intermediates, and extracellular matrix breakdown products (Ionita et al., 2010). Finally, viral-derived molecules and host nucleic acids are sensed by nucleic acid-sensing TLRs localized in the endosomal compartment, i.e., TLR3, TLR7-9 (Takeuchi and Akira, 2009). Upon ligand binding, TLR dimerization activates common signaling pathways via the adaptor MyD88, except TLR3 that uses the adaptor TRIF, leading to the induction of either pro-inflammatory molecules through the activation of NF-κB or antiviral molecules via interferon regulatory factor (IRF) routes (Figure 1).

TLR-mediated inflammation is necessary for homeostasis and control of tissue damage; however, improper activation leads to chronic inflammation, thus promoting deleterious effects. A significant amount of evidence, including human genetic studies, supports the involvement of TLR in a variety of diseases such as sepsis, asthma and autoimmune diseases (Drexler and Foxwell, 2010; Netea et al., 2012). In the cardiovascular system, recent studies support the emerging role of TLR in inflammatory diseases like atherosclerosis and ischemia/reperfusion injury (Mann, 2011), and more recently CAVD (Mathieu et al., 2015).

## TLR and CAVD pathogenesis

The finding of pathogen cargo in stenotic aortic valve cusps prompted researchers to investigate the putative role of TLR in CAVD. Although a straightforward association between infective agents and valve calcification is lacking, *Chlamydia pneumoniae* and bacteria associated with chronic periodontal infection have been detected in stenotic valves at the level of the valvular fibrosa (Juvonen et al., 1997; Nakano et al., 2006; Skowasch et al., 2009; Edvinsson et al., 2010), and inoculation of oral bacteria has been found to cause aortic valve calcification in a rabbit model of recurrent low-grade endocarditis (Cohen et al., 2004). However, the potential pathogenic role of transient bacteremia associated with mucous membrane trauma and/or the ensuing cytokine response has not been translated into clinical practice.

Innate receptors have been posited as the molecular hubs between pathogen-derived molecules, inflammation and CAVD (Mathieu et al., 2015). Particularly, the association between TLR and CAVD is supported by around 30 articles conducted in cellular and animal models. This section summarizes current evidences supporting the key role of TLR in driving inflammation and valve calcification in response to several stimuli. In the aortic valve, TLR are present not only in infiltrating immune cells but also in resident cells, where TLR4 is the most abundant subtype (Meng et al., 2008; Yang et al., 2009; López et al., 2012). To note, most evidences are from aortic valve interstitial cells (VIC), a model used to study aortic valve inflammation and calcification (Mohler et al., 1999; Osman et al., 2006; Rutkovskiy et al., 2017) that shows a myofibroblast-like phenotype when cultured on plastic plates, a reason why they are termed activated VIC or aVIC (Liu et al., 2007; Wang et al., 2014).

### TLR2/4 via MyD88/NF-κB dependent routes

The first members of the TLR family found to be expressed in aortic valve tissue and VIC were TLR4 and TLR2 (Meng et al., 2008), which are up-regulated in VIC explanted from calcified valves (Yang et al., 2009; López et al., 2012). In healthy human VICs, receptors are functional and mediate their effects via the MyD88 adaptor and the NF-κB route (Meng et al., 2008). Notably, the association of TLR with CAVD pathology has been established by using several pathogen-derived as well as endogenous molecules along the last decade (Figure 1).

#### PAMPs and nonsterile inflammation

Several highly conserved motifs present in pathogens but not in the host have been associated with inflammation and osteogenesis in the aortic valve via TLR.

Meng et al., first reported that the archetypal TLR4 agonist LPS acts as a proinflammatory factor in healthy human VIC by triggering NF-κB signaling and the subsequent induction of cytokines and adhesion molecules (Babu et al., 2008; Meng et al., 2008). These findings were later confirmed by our group and expanded to prostanoids (López et al., 2012). Moreover, VICs from calcified human valves are more responsive to LPS (Yang et al., 2009; Fernández-Pisonero et al., 2014). Remarkably, TLR4/2 ligands are also pro-calcific factors, as judged from several evidences emerged from bovine, porcine, and human VIC, as well as from mice models. Microarray and proteomic analysis revealed that LPS promotes a pro-calcific phenotype in human and clonal bovine VICs (Babu et al., 2008; Meng et al., 2008; Bertacco et al., 2010), where it functions as a pro-calcific factor by activating osteogenic mediators such as bone morphogenetic protein-2 (BMP-2), Runt-related transcription factor 2, and alkaline phosphatase. Moreover, LPS promoted *in vitro* calcification in studies using either β-glycerophosphate (Yang et al., 2009; López et al., 2012) or elevated phosphate levels (Rattazzi et al., 2008) as a phosphate source. In keeping with this, TLR2 ligands such as peptidoglycan, characteristic of the outer membrane of gram-positive bacteria, and synthetic peptides like Pam3CSK4 promote a pro-calcific phenotype in VIC (Yang et al., 2009; López et al., 2012). Even more, recent data highlight the *in vivo* role of TLR4/2 in osteogenesis since LPS promotes early aortic valve leaflet thickness increase in mice, and TLR4/2 deficiency abrogates high fat diet-induced aortic valve lesions. (Zeng et al., 2017). Interestingly, the response to TLR4/2 ligands differs according to cardiac valve site, being their effects stronger in aortic VICs as compared to pulmonary, mitral or tricuspid VICs (Yang et al., 2009; López et al., 2012; Venardos et al., 2014). Finally, a recent report hypothesizes that TLR-mediated effects are prevented in infants by a protective mechanism involving STAT3 activation that is absent in adults (Deng et al., 2015).

Together, evidences disclose a cardiac valve site-specific association between TLR4/2-NF-κB, inflammation and osteogenesis, and support an inflammation-driven calcification model in CAVD. Further studies on quiescent VIC cultured within 3D hydrogels (Hjortnaes et al., 2016) and on mice models to test the *in vivo* relevance are warranted. To note, additional LPS endosomal recognition via TRIF and TLR4-independent mechanisms cannot be ruled out (Tan and Kagan, 2014).

#### DAMPs and sterile inflammation

Endogenous molecules released upon tissue injury induce and perpetuate the so-called sterile inflammation (Ionita et al., 2010). Here, we describe several DAMPs recently linked to CAVD pathogenesis that mediate their effects via TLR.

Biglycan (BGN) is a small proteoglycan widely distributed within tissues that appears to be dysregulated in pathological conditions (Schaefer and Iozzo, 2008), including its overexpression in valves from CAVD patients (Derbali et al., 2010). Moreover, soluble BGN acts as a pro-inflammatory inducer through TLR pathways in human VICs by inducing lipid-modifying enzymes and cytokine expression via TLR2/4 (Derbali et al., 2010; Song et al., 2014). Song and colleagues further reported its pro-inflammatory and pro-osteogenic activities via a mechanism involving TLR2 and MAPK/ERK signaling (Song et al., 2012, 2014). A follow-up study demonstrated BGN-mediated pro-osteogenic reprogramming in VIC and identified BMP-2 and transforming growth factor-β1 as the molecular mediators (Song et al., 2015).

HMGB1 is a regulatory nuclear protein that when secreted extracellularly acts as a pro-inflammatory cytokine (Yang et al., 2005). Recent studies in patients and animal models have associated this protein with CAVD, since tissue and plasma levels of HMGB1 are increased in patients with CAVD (Wang et al., 2016a), and can be detected in the secretory granules of endothelial and interstitial cells explanted from diseased valves (Passmore et al., 2015). Additional evidences with recombinant HMGB1 show its pro-osteogenic activity by increasing osteogenic markers and calcium deposition in human VIC (Wang et al., 2016b). Moreover, the role of TLR4 and its transducers NF-κB and JNK, is supported by *in vitro* and *in vivo* studies, where HMGB1 pro-osteogenic activity was markedly decreased by gene silencing and TLR4-deficiency (Wang et al., 2016b; Shen et al., 2017).

Matrilin-2, an extracellular protein expressed in different tissues (Deák et al., 1999), accumulates in the calcific nodules of human aortic valves (Li et al., 2017). Moreover, matrilin-2 enhances osteogenic activity via TLR2/4 in VICs, as demonstrated by using both gene silencing and neutralizing antibodies, being the effects regulated by the NF-κB family of transcription factors and NFATc1 (Li et al., 2017).

Altogether, several DAMPs promote sterile inflammation and osteogenesis via TLR4/2-NF-κB routes, which warrants further investigation to test the *in vivo* relevance. Additional DAMPs associated to CAVD pathogenesis, i.e., galectin-3 and heat-shock protein (Skowasch et al., 2009; Sádaba et al., 2016) might mediate their effects through TLR signaling. It is plausible to think that the increased levels of DAMPs reported in calcified valves may act as a pro-calcific loop, thus contributing to disease progression.

### TLR3 and the TRIF/IRF dependent routes

The TLR3 ligand dsRNA is present in virus and can also be produced under replication of positive-strand RNA viruses, dsRNA viruses, and DNA virus, i.e., poliovirus, coxsackievirus, and encephalomyocarditis virus, in the host. An endogenous source may be tissue damage or necrosis (Gantier and Williams, 2007). The first association of viral-derived molecules with inflammation and calcification of human VIC through TLR3 was reported using polyinosinic acid: polycytidylic acid to mimic dsRNA effects (López et al., 2012). Zhan et al., later confirmed dsRNA-mediated up-regulation of inflammatory mediators and pro-osteogenic activity, and further demonstrated by gene-silencing and neutralizing antibodies the involvement of TLR3-TRIF non canonical NF-κB signaling pathways as well as the ERK route (Zhan et al., 2015, 2017). Moreover, recent *in vivo* data presented at European Society of Cardiology Congress 2017 have associated TLR3 to the onset of calcific aortic valve disease by using TLR3- and ApoE-deficient mice models (Tepekoylu et al., 2017).

In summary, these findings posit the association between TLR and osteogenesis with no reported differences between sterile and non-sterile inflammation, and support an inflammation-driven calcification model in CAVD. Future studies are needed to analyze putative mechanistic differences associated to different TLR pathways and their *in vivo* relevance.

## Modulation of TLR signaling in the aortic valve: crosstalk with membrane receptors

TLR activation relevant to CAVD can be modulated by several inflammatory mediators at various levels, but remarkably all converge on the NF-κB route. This section includes TLR modulators reported to date in the context of aortic valve physiopathology (Figure 1).

### Lipoproteins and lipid components

Among CAVD risk factors are high levels of circulating oxidized LDL (ox-LDL), which also accumulate in diseased aortic valves (Côté et al., 2008; Yeang et al., 2016). oxLDL treatment of human VIC modulates LPS effects by synergistically increasing pro-osteogenic genes (Zeng et al., 2014). Pharmacological and gene silencing approaches revealed a mechanism involving the modulation of the Notch-NF-κB axis (Zeng et al., 2014). Remarkably, blocking TLR2/4 with neutralizing antibodies abolished oxLDL-induced activation of NF-κB and ERK1/2 (Zeng et al., 2017). Sphingosine 1-phosphate, a lipoprotein lipid component acting via G-protein coupled receptors, modulates TLR activation in human VICs, macrophages and aortic endothelial cells (Dueñas et al., 2008; Fernández-Pisonero et al., 2012, 2014). In healthy human VICs, TLR-sphingosine 1-phosphate receptor interplay leads to the potentiation of inflammatory, angiogenic, and osteogenic responses through NF-κB and p38/MAPK signaling (Fernández-Pisonero et al., 2014).

### Notch

Genetic studies in humans and experiments in mice models suggest the association between the transmembrane receptor Notch and CAVD (Garg et al., 2005; Nigam and Srivastava, 2009). Recent studies provide evidence of the interplay between TLR and Notch pathways on the expression of inflammatory and osteogenic mediators and highlight the importance of their cross-talk in VICs from calcified valves, which have elevated levels of Notch1 (Zeng et al., 2012, 2013). Moreover, the mechanism of TLR4-Notch1 interplay includes the modulation of NF-κB and BMP-2 through a process dependent on Notch 1 cleavage and nuclear translocation (Zeng et al., 2012, 2013).

### Cytokines

IL-37, an anti-inflammatory cytokine that mediates its effects through IL-18 receptor and suppression of NF-κB function (Dinarello et al., 2016), is expressed in human VIC and down-regulated in calcified valves (Zhan et al., 2017). Two recent studies uncovered its anti-inflammatory and anti-osteogenic functions by modulating LPS-induced responses in human VIC (Zeng et al., 2017; Zhan et al., 2017). IL-37 negatively modulates AVIC osteogenic responses to both PAMPs such as TLR4/2 agonists, and DAMPs like oxLDL, by inhibiting ERK1/2 and NF-κB activities. Remarkably, IL-37 transgenic mice are protected against early aortic valve lesions induced by prolonged exposure to proinflammatory agents such as LPS (Zeng et al., 2017). Interestingly, IL-37 anti-inflammatory effect is specific of TLR4/2 ligands, most likely by regulating the MyD88-mediated canonical activation of NF-κB (Zhan et al., 2017).

Collectively, evidences provide mechanistic insights into the crosstalk between TLR and membrane receptors relevant to CAVD. Whether this crosstalk occurs at the level of trafficking or signaling and how these multiple pathways integrate *in vivo* needs to be elucidated. Their regulation may lead to identify therapeutic targets for the suppression of valvular inflammation.

## Conclusions and perspectives

**Figure 2:**
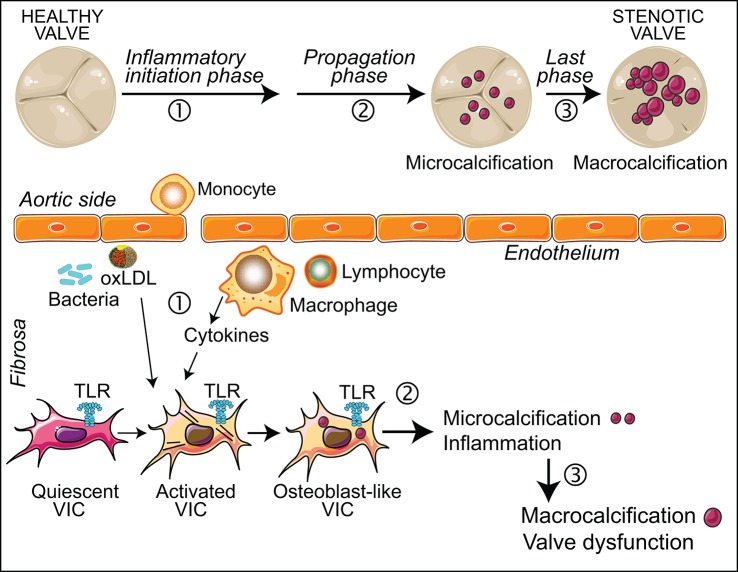
Inflammation in CAVD progression. The scheme shows the role of inflammation in the different stages of CAVD, at both valve and cellular levels. (1) Valve endothelial layer disruption leads to the recruitment of immune cells and oxLDL. This inflammatory milieu promotes the activation and differentiation of quiescent VIC. These cells express functional TLR, the activation of which induces inflammation and osteogenic reprogramming. (2) In the propagation phase, inflammation promotes the development of microcalcifications. (3) Large scale calcification leads to leaflet thickness and valve dysfunction.

Recent data highlight a potentially important link between the TLR-NF-κB axis, inflammation, and CAVD pathogenesis. Accumulating evidences suggest that TLRs could be significant contributors to the pathogenesis of valvular inflammation, and the ensuing inflammation-driven calcification processes in aortic valves (Figure 2). The key question, whether TLR signaling is involved in the onset of clinically relevant calcification, needs to be investigated using *in vivo* models recapitulating hyperphosphatemic and/or inflammatory calcification triggers, with an emphasis on the analysis of cell phenotypes to elucidate osteogenic and/or dystrophic mechanisms (Hutcheson et al., 2017). Future studies will benefit from new approaches like optical molecular imaging for the detection of early-stage calcification and 3D cultures of VIC (New and Aikawa, 2011; Hjortnaes et al., 2016), and will help to clearly define the therapeutic potential of TLRs and their signaling transducers in the initial stages of CAVD. The major challenge of targeting this pathway will be minimizing harmful innate immune responses, while preserving appropriate innate immune defense mechanisms.

## Author contributions

CG-R: Contributed to the design and the writing of the manuscript, and to figure preparation; IP-I: Drafted the manuscript and designed figures; IC-M, JL, JAS, and MS: Drafted and revised the manuscript.

### Conflict of interest statement

The authors declare that the research was conducted in the absence of any commercial or financial relationships that could be construed as a potential conflict of interest.

## Abbreviations

BGN: Biglycan
BMP-2: Bone morphogenetic protein-2
CAVD: calcific aortic valve disease
DAMP: danger/damage associated molecular patterns
HMGB1: high-mobility group box 1
IL: interleukin
IRF: interferon regulatory factor
LPS: Lipopolysaccharide
oxLDL: oxidized low-density lipoprotein
PAMP: pathogen associated molecular patterns
TLR: Toll-like receptor
VIC: aortic valve interstitial cell.
